# Supplementary material for: Safety and efficacy of N-acetylcysteine in hospitalized patients with HIV-associated tuberculosis: An open-label, randomized, phase II trial (RIPENACTB Study)
Source: PLoS One. 2020 Jun 26;15(6):e0235381. doi: 10.1371/journal.pone.0235381 (PMC7319340; doi:10.1371/journal.pone.0235381)
Supplement: S1 File — (PDF) [file pone.0235381.s003.pdf]

1. Introduction

1.1. Etiological agent and transmission

Tuberculosis (TB) is a chronic infectious disease caused by bacilli belonging to the *Mycobacterium tuberculosis* (Mtb) complex, and Mtb is the main causative agent of tuberculosis in humans (1). The spread of the disease occurs most commonly from aerosols produced by individuals infected with cough, speech and sneezing (2). Aerosol droplets containing bacillary particles are inhaled by healthy individuals and thus the bacilli lodge in the pulmonary alveoli (2). Mtb is an aerobic, immobile, unsporulated, unencapsulated bacillus measuring 1-10 micrometers long and 0.2-0.6 micrometers wide. Its cell wall is complex, formed by mycolic acids, which makes it hydrophobic, and once stained with basic dyes, cannot be bleached with alcohol and acid solution, a property called acid-fast bacillus.

1.2 Brief history of disease reception

In the nineteenth century, an idea of TB treatment emerged, where patients remained. The first questions about the sanatorium of Hermann Brehmer, a German doctor. Sanatorium comes from the word toilets, which from Latin means health, healthy (3). The sanatoriums were associated with treatment with the pure of the mountains, but underwent the dilution process and the hospitals for the treatment of tuberculosis and came to occupy a place in any climate cities. Therapy began in 1943 with the selection of para-amino-salicylic acid (PAS) and thiacetazone, which culminated in 1945 in the production of the first tuberculostatics. Both were discouraged over time because they were made exclusively

bacteriostatic. In 1944, a streptomycin, the first tuberculostatic bactericidal agent, was isolated (4). That same year, a patient who received treatment was declared cured of the disease. In 1951, an isoniazid was tested. It was followed by the development of pyrazinamide (1952), cycloserine / terizidone (1952), etionamide (1956), rifampicin (1957), capreomycin (1959) and ethambutol (1962) (5,6). With its high level of control and easy administration, a revolutionary TB treatment strategy, however, was incorporated as one shortly after its introduction. Monotherapy was discouraged and expanded as therapeutic combinations, a strategy still used today. The first analysis of tuberculosis resistance (1955-1956) involved an analysis of 974 cultures, showing a rate of 2.5% streptomycin, 2.6% for PAS and 1.3% for isoniazid (7). Subsequently, other tuberculostats appeared. Kanamycin was synthesized in 1957, and an amikacin, a semi-seventh compound derived from kanamycin, in 1972. Like fluroquinone leaves have been used since 1985 (6). However, some studies have shown interest in drug use as their own choice (8). The other Mtb medications, but were added at one dose and one bed, which are reserved for the treatment of MDR (9). The Global Health Organization (WHO) (2010) recommends that new cases be treated with the first-line regimen that is created for two months (rifagicin, isoniazid, pyrazinamide and ethambutol (RIPE) and four months of rifampicin and isoniazid). (10) The Ministry of Health (2009) adopts the same type of WHO and as of 2009 makes available for an intensive phase of fixed-dose combined tablets of the four medicines, also called 4 in 1 or Coxcip-4 (11).

1. Epidemiology

2.1. Tuberculosis in the world

It is estimated that one third of the world's culture is infected (12). In 2014, there was an increase of 9.6 million men, 3.2 million women and one million children, equivalent to 133 cases per 100,000 inhabitants. Largest in Asia (58%) and Africa (28%). India was the country that concentrated the largest number of cases (23%). Despite being a potentially curable disease, TB is among the leading causes of death from infectious disease. In 2014 alone, 1.5 million deaths occurred in the world. In the non-human immunodeficiency virus (HIV) population, 700,000 deaths were found in men, 340,000 in women and 81,000 in children. There is still a worldwide public health problem, its overall rate rate of 1.5% per year between 2000 and 2014. The mortality rate also dropped, by about 47% between 1990 and 2015 (13).

2.2. Tuberculosis in the State of Amazonas and Brazil

The state of Amazonas, located in the northern region of Brazil, is divided into 62 municipalities, with an estimated population of 3,483,985 inhabitants in 2011, 52% living in the capital, Manaus (14). Cases of TB have been reported in the Brazilian population since the 19th century, when the disease was a second leading cause of death (15). In 2012, new TB data were included in the reporting health information system (SINAN) and the TBP case rate of 67.3 / 100,000 inhabitants, when compared to the average rates of the time (36, 7 / 100,000). In 2012, the instance for bacilliferous cases was 38.8 / 100,000 inhabitants. Among the cases of pulmonary bacilliferous TB diagnosed in 2011, 78.5% are cured and 11.9% abandoned treatment. In the same year, 157 deaths were recorded, with a basic cause mortality rate of 4.4 / 100,000 inhabitants (16). From 2001 to 2011, 28,198 cases were registered in the State of Amazonas, distributed throughout all 62 municipalities. The capital Manaus concentrated the largest number of cases

(68.7%), followed by Manacapuru, Tabatinga, Itacoatiara and Tefé. There were 1,929 cases reported in indigenous peoples, a majority in communities that are a predominantly indigenous population, such as São Gabriel da Cachoeira, Itamarati and Santa Isabel do Rio Negro (14).

Brazil is part of a group of 22 WHO-prioritized countries that concentrate 80% of cases worldwide, ranking sixteenth in an absolute number of cases. Between 2005 and 2014, an average of 73,000 new TB cases were diagnosed per year. Although the year was not evaluated in any year, 67,966 new cases were diagnosed. Of these, 25,926 cases (38.1%) were registered in the Brazilian capitals. In 2014, the seventeen collections increased above the national average (33.5 / 100,000 inhabitants), especially Porto Alegre (99.3 / 100,000 inhabitants), Cuiabá (98.7 / 100,000 inhabitants), Manaus (93.8 / 100,000 inhabitants). 100,000 inhabitants), Belém (83,2 / 100,000 inhabitants) and Rio de Janeiro (82 / 100,000). The State of Amazonas and Rio de Janeiro (68.4 and 60.9 / 100,000, respectively). No reduction in debt calculation in the last two cases, in 2014, especially the State of Amazonas (15.9%), with Manaus having one of the highest dropout rates (18%) (17).

2.3. Tuberculosis and HIV

After the onset of Acquired Immunodeficiency Syndrome (AIDS), there was a worldwide increase in the number of TB cases. HIV is the main predisposing factor for the development of TB. The risk of latent form progression is 20 to 37 times higher in HIV-positive (18). In 2014, of the 9.6 million who developed TB worldwide, 1.2 million (12%) had the virus. Africa accounted for 74% of cases. In some parts of the continent, more

than 50% were co-infected. Globally, in 2014, 51% of TB patients had a documented HIV test (13). In Brazil, of the 67,966 new cases in 2014, 62.7% were tested for HIV, resulting in 10.4% of TB / HIV co-infection. The highest percentages were in Porto Alegre (28%), Curitiba (22%), Florianópolis (20.4%) and Manaus (20%) (18). For example, it is a test for tuberculosis treatment (2011) aimed at all patients with tuberculosis, poisoning, cold and bad breath (19).

A study conducted in Manaus, Amazonas, Brazil, was a leading cause of death (28%) among 129 autopsied AIDS patients (20). Janneke et al. (2010) reviewed studies that autopsied the HIV-positive series in sub-Saharan Africa over the past two decades, totaling 593 adult autopsies. Infectious diseases were the main post mortem diagnosis, being a more common TB ranging from 20-54% (21). In 2013, 360,000 co-infected people died worldwide, 50% women, making TB one of the leading causes of death in women with reproductive age HIV (12). In 2014, it is estimated that there were 385,000 deaths, 14% of children (13). Therefore, TB remains one of the leading causes of death in people with AIDS, especially in underdeveloped or socio-economic developing countries.

3. Pathophysiology of tuberculosis

After infection of infectious particles containing living bacilli, these are passed along the trachea, bronchi, bronchioles and reach the alveoli. Along the airways, a respiratory mucosa triggers the first line of defense. The alveoli are formed by type I and II epithelial cells, macrophages, neutrophils and dendritic cells (22). Macrophages are important phagocytes in an initial (or innate) immune response to Mtb. Bacillus phagocytosis

involves the participation of different types of receptors and data in intracellular environment, a sequence of events are triggered in the final phase of the process of creating a microorganism. Among these events, we can highlight: the fusion of phagosome with lysosomoma enables the proteolytic pH enzymes to act on the microorganism; production of antimicrobial chemicals such as hydrogen peroxide (reactive oxygen intermediates) and nitric oxide (nitrogen reactive intermediates), toxic metals such as iron, zinc and copper; and cell death by apoptosis (23). The response is immune to the microbiological capacity of phagocytes and Mtb virulence (24).

The dentitic cells, after bacillus phagocytosis, migrate at the site to closer lymph nodes, present Mtb antigenic peptides to CD4 + T lymphocytes, through the expression of the class II complex histocompatibility receptor (MHC) (25). This is a cellular version that represents a late (or acquired) response option that causes lymphocytes to migrate from lymph node tissue to the lung parenchyma, helping phagocytes to present (26). Macropagyos are antigen presenting strains, but this process occurs without pulmonary alveolus. The control of Mtb, mainly the success of this interaction. The CD4 + T lymphocyte deficit in AIDS shows a cell present in tuberculosis control. Proteins are required for the production of cytokines, which are a function of modulating the action of immune cells. They influence naive CD4 + T lymphocytes to differentiate into different strains. An interleukin (IL) 12 produced by phagocytes induces a production and production of Interferon Gamma (IFN-γ), which is a major cytokine involved in the Mtb immune response. Other cells may produce IFN-γ, but these lymphocytes are the main producers. In contrast, IL-4-induced lineage inhibits IFN-γ production and consequently phagocyte activation (24). When the Th1 immune response predominates the formation of granulomas occurs, which are cell-fighting in the content of spreading the bacilli,

which is not wirelessly eradicating them, which features a latent TB (27). If immune surveillance fails, the disease can be reactivated and disseminated. The bacilli tend to concentrate in the center of the often necrotic granuloma and contain macrophages surrounded by fibroblasts, neutrophils, dendritic cells and lymphocytes. Necrosis is called caseosa, from the Latin caseum, which means cheese (28). Although granuloma is intended to contain the spread of the bacillus, some studies suggest that the caseous necrosis environment may be propagated to a multiplication of Mtb (29).

Interference with host defense mechanisms is crucial for the survival of Mtb in the intracellular environment. Some people are able to adapt to the response by escaping host strategies (30). For example, an inhibition of fusion with lysosome represents one of the main mechanisms of bacillus escape from lysosomal hydrolases (31). This block allows the stability of intracellular results and prevents the presentation of antigens necessary for lymphocyte activation (32). Mtb can also counteract a production of reactive oxygen intermediates (33). Other of these mechanisms, one of the late response evocation tactics is the stimulation of IL-10 production by CD4 + T lymphocytes, tending to a Th2-type response, where an IFN-γ secretion is inhibited, favoring Mtb (34).

The different manifestations of TB reflect the balance between Mtb and host defense networks (24). In 90% of infected people Mtb is contained in the latent form (35). The spread and elimination of the bacilli is not, first of all sufficient, a lymphatic spread to the satellite lymph node, from which there may be a hematogenous spread, causing extrapulmonary forms of TB. A more common clinical manifestation is lung disease. Classical complaints may be dry or sputum, haemoptysis, fever, night sweats, asthenia

and weight loss. Extrapulmonary tuberculosis can occur in any case and follow-up is local (36).

3.1. The role of glutathione and N-acetylcysteine

Glutathione is a tripeptide synthesized by the media from cysteine sequential chain to acid glutamic, followed by addition of glycine (γ-glutamylcysteinylglycine). The cysteine sulfhydryl group (-SH) is the active componente. This tripeptide is present in most cells of the human organism and occupies the functions of functions, among them the maintenance of the cellular pathway. One of the mechanisms used to measure intracellular mortality of microorganisms is the production of oxidants such as reactive oxygen and nitrogen intermediates. Also important for this process is a simultaneous synthesis of antioxidants, a process of protection such as host cells against the effects of this bactericidal mechanism. Glutathione is one of the most important antioxidants produced by the host. The imbalance between oxidants and antioxidants is called oxidative stress, leading to decreased levels of glutathione and its precursors (38,39). Its action on system modulation has also been launched, particularly on lymphocytes. Low levels of glutathione may inhibit as cytokines in a Th1 response and induce apoptosis of CD4 + T lymphocytes (40, 41).

Unlike some antioxidants, they can also be reverted to a synthesis of glutathione (39). Also, the administration of glutathione is not aerogenized due to its bioavailability and its limited ability to cross a phospholipid bilayer of cells. Similarly, a cysteine undergoes rapid oxidation to its disulfide, cystine, which has poor solubility. N-acetylcysteine (NAC) first made its report during the 1960s, when it proved to be an effective mucolytic

agent in individuals with cystic fibrosis. Subsequently, a new role in investigating its therapeutic potential in acetaminophen poisoning. Cleavage of the acetyl group makes cysteine available for later adoption in glutathione synthesis (Figure 1), decreased in the hepatic reaction by acetaminophen action. The effect causes NAC to have an indirect antioxidant effect, which aroused in the 1980s an interest in studying its effect on diseases that undergo oxidative stress (42). Different lines of research addressing the topic as in the pathogenesis of chronic obstructive pulmonary disease (COPD). PANTHEON has recently proposed a NAC as a complement to COPD treatment, with daily doses of 1,200 mg of compound (43).

TB and AIDS are also diseases that develop with chronic stimulation, with a rapid formation of free radicals that, in excess, can generate cellular and systemic oxidative stress. NAC are just a few as they are studied. Some authors have seen that with TB there are glutathione sublevels (44, 45, 46). The same occurred in a model of Mtb-infected animals, suggesting that oxidative stress was due in part to a poor antioxidant host defense. NAC supplementation decreases the bacterial burden on the chest and the severity of non-lung necrosis (47). Kaiser *et al*. (2010) argue that equilibrium enters as an oxidizing and antioxidant substance in a critical role in inducing IL-12 production involving a Th1 response and that the use of NAC may be useful for better immune control of TB (48). Additional benefits of NAC in case of contraception alongside tuberculostatic (49) and the direct antimicrobial effect demonstrated in vitro. Amaral et al. (2016) observed that NAC treatment in Mtb cultures reduced a bacterial metabolic activity after the experiment period. These cases suggest that a NAC has a direct bacterial action without growth of the bacillus (without press).

Reduced glutathione levels are also measured in AIDS patients (50). Guerra et al. (2011) demonstrated that HIV-negative levels have CD4 + T lymphocyte glutathione levels when compared to healthy control, and these levels are restored after in vivo supplementation with NAC, favoring cytokine production in a Th1 response. We also observed that glutathione depletion in lymphocytes correlated with one of the levels of tumor necrosis factor (TNF) and free radicals. TNF is a proinflammatory cytokine associated with the progression of HIV and the maintenance of inflammatory damage (50). The reactive energies of oxidized oxygen are involved in the activation of nuclear factor-kB (NF-kB), which controls gene transcription for HIV replication (51). Vishwanath et al. (2006) suggested that the immune system's inability to high rates of HIV-positive (or not) compared to the maximum glutathione levels in macrophages (53). Morris et al. (2013) documented that the recovery of glutathione levels reversed the loss of innate immunity functions, which is a new control mechanism of antiretroviral treatment (54).

There are still data in the literature with the use of NAC in the population of co-infected TB / HIV are limited, it is a safe example, with the possibility of low cost side effects. The hypothesis of its response is Th1 immune and has a direct antimicrobial action against HIV, which justifies this study. This proposal has become more possible when you built the effect of hepatoprotectant on mycobacteriostats.

4. Objectives

4.1. General objective

To evaluate the effects of NAC in individuals coinfected with HIV;

4.2. Specific objectives

• Evaluate the safety, tolerance and effectiveness of adjuvant therapy in the treatment of tuberculosis;

• Observe smear and culture conversion rates for mycobacteria in the first 8 weeks of NAC use;

4. Methods

4.1. Study Design

RIPENACTB is a phase II, randomized, controlled, open, single-group, parallel clinical trial.

4.2. Study site

The study will be conducted at the Tropical Medicine Foundation, in the city of Manaus, capital of the state of Amazonas. FMT-HVD is an institution of the Government of the State of Amazonas, created in 1974, which has as national and international reference the medical assistance, teaching and research in infectious and parasitic diseases. The complex is divided into inpatient unit, outpatient unit, clinical analysis laboratories (including TB laboratory, security level III) and research management.

4.3. Participants

4.3.1. Eligibility Criteria

4.3.1.1. Patients admitted to ICU, wards or emergency room

- Age greater than or equal to 18 years;
- Acceptance of HIV testing;
- Suspected pulmonary TB;
- Estimated hospital stay over 24 hours;
- Consent signed and dated prior to initial assessment.

4.3.2. Exclusion Criteria

4.3.2.1. For the use of NAC

- Indigenous;
- Refusal to perform the HIV test;
- Pregnant women, nursing mothers or recording plans during the study period;
- Extra pulmonary TB, without pulmonary involvement;

The conditions of home collection of sputum or tracheal aspirate for mycobiological, even if induced;

- No positive culture for Mtb;
- Mono, poly or multiresistance to Mtb, detected in the sensitivity profile;

- Individuals undergoing treatment for bronchospasm, a bronchial asthma, as decided by the study assistant or researcher;
- Clinical suspicion of gastric or duodenal ulcer, with evaluation of medical records or study researcher; upper digestive endoscopy;
- Alanine aminotransferase (ALT) greater than three times normal;
- Need to suspend the RIPE scheme, under consultation of the researcher;
- Missing according to treatment for more than seven consecutive days;
- Inability to follow up;
- Withdrawal of consent.

There is therefore a reason for withdrawal statement. The same should happen in the two FMT-HVD specialized outpatient clinics to continue the treatment of TB and HIV.

4.4. Recruitment and follow-up

4.4.1. Via tuberculosis laboratory

The FMT-HVD tuberculosis laboratory will be oriented to conduct a series of studies on the positivity of some test for TB, regardless of the biological material analyzed. The physician should investigate the patient's procedure and obtain information necessary to analyze a possibility of inclusion in the study.

4.4.2. Via intensive care unit visits, wards and emergency care

The study team will make daily visits to these groups, along with the care team, for eligibility issues.

4.4.3. Eligible Individuals

If the candidate is eligible, he/she will be invited to participate in the RIPENACTB study through the informed consent form, as determined by the researcher. Unable to understand, IC will be offered to the nearest family member. To be accepted, a proof collection of the RIPENACTB protocol should be performed as needed. There will be an attempt to match a study collection with a technical assistance collection, with the intention of reducing the number of punishments. In the presence of central venous access, a medical record will be aspirated from this device. The results of the RIPENACTB protocol are not D0, occurrences obtained, are valid if they obey the following tolerance intervals:

- Beta-hcg (urine or blood): up to seven days;
- Blood count, urea, aspartate aminotransferase (AST), alanine aminotransferase (ALT), bilirubins, total proteins and fractions: up to 24 hours;
- Rapid (serological) test for HIV, hepatitis B and C: for six months;
- HIV viral load and CD4 + / CD8 + count: up to six months;
- posterolateral chest radiograph (PA) and chest profile or tomography: up to seven days;

The hospital discharge, the elderly is accompanied by the study team, which occupies the infectious disease doctor and nurse through the RIPENACTB outpatient clinic,

present in the clinical research ward (PESCLIN) of FMT-HVD. Study consultations are scheduled in the following and the following:

- D0: eligibility, medical consultation, exam collection and medication release (RIPE / RIPE + NAC);
- D7 (week 1): exam collection;
- D14 (week 2): medical consultation, exam collection, introduction of ART, following the norms of the Ministry of Health and Public Health (RIPE / RIPE + NAC);
- D28 (week 4 / month 1): medical consultation, exam collection and medication release (RIPE / RIPE + NAC);
- D42 (week 6): medical consultation, exam collection and medication release (RIPE / RIPE + NAC);
- D56 (week 8 / month 2): medical consultation, exam collection and completion of the intensive phase of NAC and medication release (R + I);
- Month 4: nursing consultation, exam collection and medication release (R + I);
- Month 6: medical consultation, examination form, evaluation of tuberculosis cure and study participation in the study.

In case of absence, a tolerance of seven days will be given for rescheduling, otherwise it will be considered as impossible to follow up. Babies are advised to come up with a medical indication. Upon completion of TB and FDA treatment, they will be referred to the FMT-HVD HIV Outpatient Clinic for follow-up.

4.4.4. Collection of sputum or tracheal aspirate

The material will be delivered to the FMT-HVD TB lab by the study nurse. As the basins must be subjected to bacilloscopy, Xpert MTB / RIF® (diagnostic only) and culture for continuous liquid mycobacteria. Should be processed within one hour and should be oriented to data processing and reported for the following cases: not identified or misidentified; present leakage; or inappropriate pool. The antimicrobial susceptibility test will be done on sample D0, which is being observed Mtb.

5. Interventions:

5.1. Study Treatments

Group A: RIPE therapy: Rifampicin 150 mg + isoniazid 75 mg + pyrazinamide 400 mg + ethambultol 275 mg (combined fixed dose tablet) for 2 months.

Group B: RIPE and NAC therapy: Rifampicin 150 mg + isoniazid 75 mg + pyrazinamide 400 mg + ethambultol 275 mg (combined fixed dose tablet) and NAC 600mg bid, for 2 months.

NAC is a drug approved by the United States Food and Drug (FDA) and ANVISA, category B in pregnancy. When administered orally it is easily absorbed by the intestinal mucosa. In the liver, it is converted to cysteine to form glutathione, which is then secreted into the circulation. Its half life is 5.6 hours and approximately 30% is excreted by the kidneys. Nausea, vomiting and heartburn may occur, but side effects are uncommon (55). Oral administration of NAC at doses up to 8000 mg / day did not lead

to clinically significant adverse reactions (56). Interaction with tuberculostatics is not known.

5.2. Medication storage and handling

Study drugs will be stored in a safe place and under appropriate physical conditions. Access will be limited to the researcher and authorized staff. The release will be made during medical consultations registered in the source file "idoctor".

5.3. Duration of treatment

The use of NAC and the RIPE scheme will be for two months (intensive phase). The use of rifampicin and isoniazid (maintenance phase) cannot be pre-established because, although this phase lasts an average of four months, it can be extended as directed by a physician.

5.4. Dosage and administration

The RIPE regimen will be administered orally or nasogastric tube via the 4 in 1 tablet, with the dose adjusted for weight once a day and preferably for fasting. NAC will be administered orally or via a nasally sachet-shaped effervescent sachet at a dose of 1200 mg (two 600 mg sachets) to be diluted in 200 ml of water and administered once daily.

5.4. Treatment group

The examples will be assigned to treatment according to a randomization table.

5.5. Medication Accounting

The researcher should document in the medical records the amount of medicines dispensed, administered and administered by a case, returned. Accounting records should be updated until treatment ends.

5.6. Treatment adherence

They will be charged for medical appointments such as dispensing medication packages and first-time telephone operators once a week, during working hours, or mandatory SMS or WhatsApp® messages to emphasize adherence to treatment.

5.7. Concomitant medications and non-drug therapies

All concomitant medications known during the study should be done in a medical record. There is no indication for concomitant use of any medications.

5.8. Treatment after study termination

Not much planned extension time. Upon completion of TB treatment, doctors will be revoked at the FMT-HVD HIV / AIDS outpatient clinic.

5.9. Overdose treatment of the investigational drug

An overdose for this study will be calculated as any dose higher than planned. So far there is no one specific to the NAC. Tax on the adverse effect due to overdose of the NAC, the object must be managed with supportive measures and may be hospitalized in PESCLIN, receiving the care of the researcher. After hospital discharge, the main responsible is to return to the tuberculosis treatment routine, in addition to being able to return to treatment.

5.10. Adverse and suicidal events in research (NAC)

The classification of adverse events will be based on the AIDS Division (DAIDS) Severity Rating Table for Adverse Events in Adults and Pediatrics (Annex 1), in a journal published in HIV clinical trials. The DAIDS leaderboard has a severity scale ranging from one to five.

- Grade 1: mild event;
- Grade 2: Moderate event;
- Grade 3: serious event;
- Grade 4: potentially fatal event;
- Grade 5: death.

In the presence of a four- or four-year adverse or laboratory problem, researcher discontinuity will be judged by the researcher, as the adverse event must be reported by an external control committee within 24 hours. The chance of being recorded in the patient's medical record must be related to TB treatment.

6. Outcomes

6.1. Primary work

A safety, tolerability and action of the NAC.

6.2. Secondary Outcomes

The converse to conversion of bacilloscopy and the culture in the weeks.

Monitor drug hepatotoxicity to tuberculostatics.

To evaluate the effect of NAC on viral load and CD4 + cell count.

7. Sample Size and Randomization

To not fifty evidence should be planned to be randomized (twenty five in each group), using the table of the program Excel®.

8. Statistical Analysis

Data will be entered in an Excel® 8.0 spreadsheet and statistical analysis will be performed using the Stata 11.0 statistical program. For a descriptive analysis: 1) means and standard deviation are calculated for continuous variables with normal distribution; 2) medians and interquartile ranges (IQI, 25-75% percentile) for continuous asymmetric distribution variables; 3) proportions for categorical variables. In the bivariate exploratory investigation the chi-square hypothesis test or Fisher's exact test will be applied to the

variable variables and the continuous distributed variables will be analyzed with the Student test in the media communication, with or without adjustment of unequal variances. Continuous variables Asymmetry distribution or variables are analyzed using the nonparametric Mann-Whitney or Kruskal-Wallis test to compare the medians. The measure used to calculate the difference between risk variables (RR) and explanatory variables (covariates of interest). Multivariate will be detected by logistic regression model, with derivation of odds ratio (OR or odds ratio). Confusion will be analyzed through non-automatic editing of variables back and forth one by one (backwards, step by step). It will be a protocol data analysis (PP) and an intention-to-treat analysis (ITT). Confidence interval of 95% (95% CI) and importance values will be considered when less than 0.05. They will be a preliminary investigation when reaching the follow-up of half of the patients to be randomized.

9. Ethical Considerations

This study will be submitted to the FMT-HVD Research Ethics Committee (CEP), preserving the rights of the research subjects.

12. Financing

A NAC will be purchased by by INTERFAM. Reference therapy was no longer flat because it is being advocated as first-line TB treatment by the Ministry of Health.

13. Comments on the feasibility and implementation of the proposal

For the feasibility and execution of the first study shift, data selection is available for the study; financing for a purchase of consumables; eligible patients, as they are a national and international referral center for infectious and parasitic diseases; and partnership with researchers in the area.

14. Conflicts of Interest

There is no conflict of interest in the development of this study.

References

1. Frieden TR, Sterling TR, Munsiff SS, Watt CJ, Dye C. Tuberculosis. Lancet. 2003;362(9387):887–899.

2. Lawn SD, Zumla AI. Tuberculosis. Lancet. 2011;378(9785):57–72.

3. Daniel TM. Hermann Brehmer and the origins of tuberculosis sanatoria. Int J Tuberc Lung Dis. 2011;15(2):161-162.

4. Daniel TM. The history of tuberculosis. Resp Med. 2006;100(11):1862-1870.

5. Keshavjee S, Farmer PE. Tuberculosis, drug resistance, and the history of modern medicine. NEJM. 2012;367(10):931-936.

6. Arbex MA, Varella Mde C, Siqueira HR, Mello FA. Antituberculosis drugs: drug interactions, adverse effects, and use in special situations. Part 2: Second line drugs. J Bras Pneumol. 2010;36(5):641-656.

7. Fox W, Wiener A, Mitchison DA, Selkon JB, Sutherland I. The prevalence of drug-resistant tubercle bacilli in untreated patients with pulmonary tuberculosis: a national survey, 1955-56. Tubercle. 1957;38(2):71-84.

8. Ziganshina LE, Titarenko AF, Davies GR. Fluoroquinolones for treating tuberculosis (presumed drug-sensitive). Cochrane Database Syst Rev. 2013;6(6):1-83.

9. Zumia A, Chakaya J, Centis R, D'Ambrosio L, Mwaba P, Bates M, et al. Tuberculosis treatment and management-an update on treatment regimens, trials, new drugs, and adjunct therapies. Lancet Respir Med. 2015;3(3):220-234.

10. WHO. The treatment of tuberculosis: guidelines. Geneva: World Health Organization; 2010.

11. Ministério da Saúde. Secretária de Vigilância em Saúde. Departamento de Vigilância Epidemiológica. Programa Nacional de Controle da Tuberculose. Nota técnica sobre as mudanças no tratamento da tuberculose no Brasil para adultos e adolescentes - versão 2. Brasília: Ministério da Saúde; 2009.

12. WHO. Global tuberculosis 2014. Geneva: World Health Organization; 2014.

13. WHO. Global tuberculosis 2015. Geneva: World Health Organization; 2015.

14. Garrido Mda S, Bührer-Sékula S, Souza AB, Ramasawmy R, Quincó Pde L, Monte RL, et al. Temporal distribution of tuberculosis in the State of Amazonas, Brasil. Rev Soc Bras Med Trop. 2015:48(1):63-69.

15. Batista D. Aspectos epidemiológicos da tuberculose no Amazonas. Bol Oficina Sanit Panam 1953;5:4333-4347.

16. Ministério da Saúde. Panorama da tuberculose no Brasil: indicadores epidemiológicos e operacionais. Brasília: Ministério da Saúde; 2014.

17. Ministério da Saúde. Secretaria de Vigilância em Saúde. Boletim epidemiológico. Detectar, tratar e curar: desafios e estratégias brasileiras frente a tuberculose. Brasília: Ministério da Saúde; 2015.

18. Granich R, Akolo C, Gunneberg C, Getahun H, Williams P, Williams B. Prevention of tuberculosis in people living with HIV. Clin Infect Dis. 2010;50(S3):S215-S222.

19. Ministério da Saúde. Manual de recomendações para o controle da tuberculose no Brasil. Brasília: Ministério da Saúde; 2011.

20. Souza SL, Feitoza PV, Araújo JR, Andrade RV, Ferreira LC. Causes of death among patients with acquired immunodeficiency syndrome autopsied at the Tropical Medicine Foundation of Amazonas. Rev Soc Bras Med Trop. 2008;41(3):247-251.

21. Cox JA, Lukande RL, Lucas S, Nelson AM, Van Marck E, Colebunders R. Autopsy causes of death in HIV-positive individuals in Sub-Saharan Anfrica and correlation with clinical diagnoses. AIDS. 2010;12(4):183-194.

22. Lerner TR, Borel S, Gutierrez MG. The innate immune response in human tuberculosis. Cell Microbiol. 2015;17(9):1277-1285.

23. Weiss G, Schaible UE. Macrophage defense mechanisms against intracellular bacteria. Immunol Rev. 2015;264(1):182-203.

24. van Crevel R, Ottenohff TM, van der Meer, JW. Innata immunity to *Mycobacterium tuberculosis*. Clin Microbiol Rev. 2002;15(2):294-309.

25. Marino S, Pawar S, Fuller CL, Reinhart TA, Flynn JL, Kirschener DE. Dendritic cell trafficking and antigen presentation in the humam immune response to *Mycobacterium tuberculosis*. J Immunol. 2004;173(1):494-506.

26. Orme IM, Robinson RT, Cooper AM. The balance between protective and pathogenic immune responses in the TB-infected lung. Nat Immunol. 2015;16(1):57-63.

27. Lin PL, Flynn JL. Understanding latent tuberculosis: a moving target. J Immunol. 2010;185(1):15-22.

28. Flynn JL, Chan J, Lin PL. Macrophages and control of granulomatous inflammation in tuberculosis. Mucosal Immunol. 2011;4(3):271-278.

29. Shaler CR, Horvath CN, Jeyanathan M, Xing Z. Within the Enemy's Camp: contribution of the granuloma to the dissemination, persistence and transmission of *Mycobacterium tuberculosis*. Front Immunol. 2013:4(30):1-8.

30. Kaufmann SH. How can immunology contribute to the control of tuberculosis? Nat Rev Immunol. 2011;1(1):20-30.

31. Rohde K, Yates RM, Purdy GE, Russel DG. *Mycobacterium tuberculosis* and the environment within the phagosome. Immunol Rev. 2007;219:37-54.

32. Poirier V, Av-Gay Y. *Mycobacterium tuberculosis* modulators of the macrophage's cellular events. Microbes and Infection. 2012;14(13):1211-1219.

33. Shin DM, Jeon BY, Lee HM, Jin HS, Yuk JM, Song CH, et al. *Mycobacterium tuberculosis* eis regulates autophagy, inflammation, and cell death through redox-dependent signaling. PLoS Pathog. 2010;6(12).

34. Grupta A, Kaul A, Tsolaki AG, Kishore U, Bhakta S. *Mycobacterium tuberculosis*: immune evasion. latency and reactivation. Immunobiology. 2012;217(3):363-374.

35. Zumla A, Raviglione M, Harner R, von Rey F. Tuberculosis. NEJM. 2013;368(8):745-755.

36. Cruz-Knight W, Blake-Gumbs. Tuberculosis: An Overview. Prim Care. 2013;40(3):743-756.

37. Ghezzi P. Role of glutathione in immunity and inflammation in the lung. Int J Gen Med. 2011;4:105-113.

38. Venketaraman V, Dayaram YK, Amin AG, Ngo R, Green RM, Talaue MT, et al. Role of glutathione in macrophage control of mycobacteria. Infect Immun. 2003;71(4):1864-1971.

39. Atkuri KR, Mantovani JJ, Herzenberg LA, Herzenberg LA. N-acetylcysteine - a safe antidote for cysteine/glutathione deficiency. Curr Opin Pharmacol. 2007;7(4):355-359.

40. Peterson JD, Herzenberg LA, Vasquez K, Waltenbaugh C. Glutathione levels in antigen-presenting cells modulate Th1 versus Th2 response patterns. Proc Natl Acad. 1988;95(6):3071-3076.

41. Gil L, Martínez G, González I, Tarinas A, Álvarez A, Giuliani A, et al. Contribution to characterization of oxidative stress in HIV/AIDS patients. Pharmacol Res. 2003;47(3):217-224.

42. Rushworth GF, Megson IL. Existing and potential therapeutic uses for N-acetylcysteine: the need for conversion to intracellular glutathione for antioxidant benefits. Pharmacol Ther. 2014;141(2):150-159.

43. Zheng JP, Wen FQ, Bai CX, Wan HY, Kang J, Chen P, et al. Twice daily N-acetylcysteine 600 mg for exacerbations of chronic obstructive pulmonary disease (PANTHEON): a randomised, double-blind placebo-controlled trial. Lancet Respir Med. 2014;2(3)187-194.

44. Vijayamalini M, Manoharan S. Lipid peroxidation, vitamins C, E and reduced glutathione levels in patients with pulmonary tuberculosis. Cell Biochem Funct. 2004;22(1):19-22.

45. Reddy YN, Murthy SV, Krishna DR, Prabhakar MC. Role of free radicais and antioxidants in tuberculosis patients. Indian J Tuberc. 2004;51:213-218.

46. Venketaraman V, Millman A, Salman M, Swaminathan S, Goetz M, Lardizabal A, et al. Glutathione levels and immune responses in tuberculosis patients. Microb Pathog. 2008;44(3):255-261.

47. Palanisamy GS, Kirk NM, Ackart DF, Shanley CA, Orme IM, Basaraba RJ. Evidence for oxidative stress and defective antioxidant response in guinea pig with tuberculosis. PLoS One. 2011;6(10)1-13.

48. Alam K, Ghousunnissa S, Nair S, Valluri VL, Mukhopadhyay S. Glutathione-redox balance regulates c-rel-driven IL-12 production in macrophages: possible implications in antituberculosis immunotherapy. J Immunol. 2010;184(6):2918-2929.

49. Baniasadi S, Eftkhari P, Tabarsi P, Fahimi F, Raoufy MR, Masjedi MR, et al. Protective effect of N-acetylcysteine on antituberculosis drug-induced hepatotoxicity. Eur Gastroenterol Hepatol. 2010;22(10):1235-1238.

50. Guerra C, Morris D, Sipin A, Kung S, Franklin M, Gray D, et al. Glutathione and adaptive immune responses against *Mycobacterium tuberculosis* infection in healthy and HIV infected individuals. PLoS One. 2011;6(12):1-9.

51. Schreck R, Rieber P, Baeuerle PA. Reactive oxygen intermediates as apparently widely used messengers in the activation of the NF-kappa B transcription factor and HIV-1. EMBO J. 1991;10(8):2247-2258.

52. Staal FJ, Roederer M, Herzenberg LA, Herzenberg LA. Intracellular thiols regulate activation of nuclear factor kappa B and transcription of human immunodeficiency virus. Proc Nati Acad Sci USA. 1990;87(24):9943-9947.

53. Venketaraman V, Rodgers T, Linares R, Reilly N, Swaminathan S, Hom D, et al. Glutathione and growth inhibition of Mycobacterium tuberculosis in healthy and HIV infected subjects. AIDS Res Ther. 2006;3(5):1-12.

54. Morris D, Guerra C, Khurasany M, Guilford F, Saviola B, Huang Y, et al. Glutathione supplementation improves macrophage functions in HIV. J Interferon Cytokine Res. 2013;33(5):270-279.

55. Sansone RA, Sansone LA. Getting a Knack for NAC: N-Acetyl-Cysteine. Innov Clin Neurosci. 2011;8(1):10-14.

56. De Rosa SC, Zaretsky MD, Dubs JG, Roederer M, Anderson M, Green A, et al. N-acetylcysteine replenishes glutathione in HIV infection. Eur J Clin Invest. 2000;30:915–929.
