# Supplementary material for: Safety and efficacy of N-acetylcysteine in hospitalized patients with HIV-associated tuberculosis: An open-label, randomized, phase II trial (RIPENACTB Study)
Source: PLoS One. 2020 Jun 26;15(6):e0235381. doi: 10.1371/journal.pone.0235381 (PMC7319340; doi:10.1371/journal.pone.0235381)
Supplement: Attachment — Submitted filename: Rebuttal letter Safe et al 21_05_2020f.docx [file pone.0235381.s005.docx]

Dear Editor,

We appreciate the opportunity to clarify the points raised by the reviewer and believe that the manuscript has improved significantly.

Reviewer #4: A phase II randomized two-arm controlled clinical trial was conducted to assess the safety and tolerability of N-acetylcysteine (NAC) in HIV patients with tuberculosis. The adverse event rates were similar in the two arms, and no evidence was found to refute that NAC was unsafe.

Minor revisions:
1- Clarify that the adverse event RATES were similar in the two arms.
Response: The sentence was adjusted accordingly in the abstract.

2- Statistical Analysis Section:
A. Differences in categorical variables were TESTED using Fisher’s exact test.
Response: The sentence was adjusted accordingly.

B. This statement is unclear, “generalized linear regression with log binomial.” More fully explain the statistical methods associated with the general linear regression model.
Response: The sentence was rephrased to clarify this point.

“Univariate log-binomial generalized linear regression with respective 95% confidence intervals (CI) was used to estimate relative risks (RR) in order to assess associations with the major outcomes of the study”.

C. Remove “Differences with” in the sentence “Differences with p-values < 0.05 were considered statistically significant.
Response: The sentence was adjusted accordingly.

3- Replace NS with numeric p-values.
Response: Replaced accordingly.

4- Figure 2: Indicate the type of test used to generate the p-value.
Response: The following sentence was added to the graph legend: “P-values refer to RR estimates” to clarify.

5- State and justify the study’s target sample size with a pre-study statistical power calculation.

Response: A paragraph including sample size calculation was added to the manuscript Study participants section as follows: “For the sample size calculation, a percentage of 37.5% of hepatotoxicity among the RIPE group and no episodes for RIPENAC was considered [12]. A 1:1 ratio with a power of 80% and a significance level of 95% was used. A total sample size of 36 was estimated.
